# Cyberbullying victimization predicts substance use and mental health problems in adolescents: data from a large-scale epidemiological investigation

**DOI:** 10.3389/fpsyg.2025.1499352

**Published:** 2025-04-28

**Authors:** Cláudio Romualdo, Wanderlei Abadio de Oliveira, Luciana Bertoldi Nucci, José Eugenio Rodríguez Fernández, Laura Soares da Silva, Evelin Moreira Freires, Amanda Severo Lins Vitta, Fernando Ferreira Semolini, Lucas da Rosa Ferro, Denise de Micheli, André Luiz Monezi Andrade

**Affiliations:** ^1^School of Life Sciences, Pontifical Catholic University of Campinas, Campinas, Brazil; ^2^Faculty of Education Sciences, University of Santiago de Compostela, Santiago, Spain; ^3^Department of Psychobiology, Federal University of São Paulo, São Paulo, Brazil

**Keywords:** adolescents, cyberbullying, emotional issues, risky behaviors, mental health

## Abstract

**Purpose:**

This study investigated the potential association of cyberbullying victimization (CyVic) on substance use and mental health-related behaviors among Brazilian adolescents, using data derived from the National Survey of School Health (PeNSE).

**Method:**

The sample comprised 146,536 adolescents aged up to 17 years, who were selected through probabilistic and representative sampling. Participants were categorized into two groups: cyber-victim group (CYB), comprising those who reported experiencing threats, offense, or humiliation on social media platforms or messaging apps 30 days before the survey, and non-cybervictim group (nCYB).

**Results:**

Of the participants, 13.3% were classified in the CYB group, with the majority being girls (61.9%) compared to boys (38.1%). The CYB group showed a significantly higher prevalence of substance use, with 22% of these youths reporting illicit drug use compared with 11% in the nCYB group. The CYB group also exhibited significant alcohol and tobacco consumption, as well as the initiation of these substances at a younger average age compared to the nCYB group. Sadness, helplessness, anxiety, and suicidal thoughts were significantly higher in the CYB group. The CYB group also reported lower parental understanding and a higher incidence of aggression, sexual abuse, and episodes of violence among peers and family members.

**Conclusion:**

The study results revealed the negative consequences of CyVic, emphasizing the need for effective strategies to address this issue and promote adolescent mental health.

## 1 Introduction

The term “cyberbullying” is a linguistic amalgamation of the word “bullying,” signifying purposeful and repetitive acts of aggression characterized by a power disparity. The prefix “cyber,” conveys the manifestation of these behaviors within virtual domains. Cyberbullying transgressions are not haphazard occurrences, but rather deliberate assaults intended to cause harm or unease to the targeted individual (Lembo et al., 2023; Zhang et al., 2022). This antagonistic conduct adopts many forms, including harassment, derogatory remarks, threats, defamation, online exclusion, and the illicit appropriation of an individual’s identity and personal data (Menin et al., 2021). Crucially, cyberbullying entails a malevolent intent to cause harm, irrespective of the specific manifestation of aggression. A defining attribute of cyberbullying is its underlying power disequilibrium (Barlett et al., 2022). Unlike conventional bullying, in which this disparity may involve physical or social dimensions, in the digital realm, it may manifest as technological inequity—whereby the cyber perpetrator possesses advanced skills to intimidate the victim through technology—or psychological disparity, whereby the cyberbullying victim experiences sensations of fear, timidity, or heightened vulnerability (Wang and Ngai, 2022).

The United Nations Children’s Fund conducted a comprehensive study concerning the epidemiological facets of cyberbullying in more than 30 countries, with approximately 170,000 individuals aged 13–24 years, and found that nearly 30% of respondents acknowledged being cyberbullying victims at least once (U-Report, 2019). Of importance to the present study, nearly 37% of participants in Brazil reported being cyberbullying victims, and youth participants exhibited the highest prevalence of school absenteeism because of cyberbullying instigated by peers (36%). The same report underscored that cyberbullying was most prevalent on social media platforms, such as Facebook, Instagram, Snapchat, and Twitter.

In another global investigation spanning 10 nations and involving over 11,000 parents and children, 21% of juveniles and adolescents admitted to engaging in cyberbullying directed at acquaintances (McAfee, 2022), with Brazil recording a prevalence of 22%. The same study indicated that 67% of the participants had experienced discrimination from acquaintances and 51% from unfamiliar individuals. A study by the Brazilian Internet Management Committee (ICT et al. Brazil) with 2,954 children and adolescents aged 9–17 years, found that 7% reported personally experiencing cyber discrimination. By contrast, 43% had witnessed online cyber discrimination (Brazilian Internet Management Committee [BIMC], 2019). Intriguingly, online discrimination frequency directly correlates with Internet and social media engagement. Individuals accessing the Internet (49%) and social media (63%) daily reported more frequent encounters with these behaviors. By contrast, those engaging in online activities once a week, using the internet (17%) and social media (30%), displayed a diminished frequency of such incidents. In other words, there is a correlation between cyberbullying victimization (CyVic) and increased internet use, which is noteworthy for the present study; some authors have also established a correlation between CyVic and excessive smartphone usage (Qudah et al., 2019; Ramos et al., 2023; Wang and Jiang, 2022), as well as heightened Internet engagement (Albikawi, 2023; Méndez et al., 2020; Sureda Garcia et al., 2020). The augmented exposure stemming from these behaviors accentuates cyberbullying susceptibility, a fact that is especially consequential, considering that Brazil ranks third globally in the average time spent on the Internet (World Wide Web Foundation [WWWF], 2019) and second in out-of-school Internet engagement time among students (Organization for Economic Co-operation and Development [OECD], 2017).

Regarding emotional well-being, previous studies have reported a link between depression and cyberbullying. Specifically, cyberbullying victims report markedly higher occurrences of depressive symptoms and behaviors compared to non-victims (Xie et al., 2022). Similarly, cyberbullying victims experience heightened anxiety levels and chronic anxiety due to the fear of repeat victimization (Wilson et al., 2023). CyVic has also been linked to lower self-esteem, as victims frequently internalize negative messages propagated by aggressors, leading to detrimental and negative self-perceptions (Palermiti et al., 2022). Moreover, isolation further exacerbates emotional health decline; cyberbullying victims, particularly within educational settings, often experience isolation from peers, coupled with a dearth of social support from educators, parents, and other family members (Sattler et al., 2023).

Substance use has also been found to be an additional risky behavior associated with CyVic (de Oliveira Pinheiro et al., 2020). Considering the heightened susceptibility of adolescents, vis-à-vis adults, to the reinforcing properties of drugs within the reward system (Salmanzadeh et al., 2020), the adolescents are prone to developing a trajectory of drug addiction symptoms (Ernst, 2014). Previous studies have demonstrated a link between CyVic and substance use (McField et al., 2023). Given the significant distress associated with cyberbullying, individuals may turn to substance use to cope with their negative emotions (Azami and Taremian, 2020). Nevertheless, this relationship is complex and may be influenced by multiple factors. A systematic review by Biagioni et al. (2023), regarding cyberbullying in individuals aged 10 to 20 years, examined 50 studies. They found a positive correlation between cyberbullying and drug use, most notably tobacco and alcohol use. Regarding illicit substances, marijuana exhibited the most significant association with cyberbullying. Moreover, the study found that female cyber victims demonstrated a heightened propensity for alcohol consumption, smoking, binge drinking, and non-prescription medication use (e.g., benzodiazepines). Notably, low support of parental dynamics moderated this relationship. These findings suggest that parental oversight is pivotal in identifying instances of CyVic and the potential co-occurrence of substance use.

Despite the heightened rates of Internet use in Brazil, limited empirical research has examined the potential correlation between CyVic and various risky behaviors and emotional disorders. Particularly, empirical research on the relationship between cyberbullying and substance use remains non-existent in Brazil. This is a significant gap, given that, in the latest iteration of the National Survey of School Health (PeNSE) conducted by the Brazilian Institute of Geography and Statistics (Instituto Brasileiro de Geografia e Estatística [IBGE], 2022), 12.3% of adolescents reported experiencing CyVic. Notably, this survey employed a methodologically robust, representative, and probabilistic sampling strategy that encompassed the Brazilian adolescent population within a designated age range. Thus, the findings of this survey are highly generalizable to Brazilian adolescents.

Although the PeNSE is available as an open-access dataset, to date, no study has examined the psychosocial association of CyVic, including substance use, emotional aspects, familial relationships, and academic factors. Research using representative samples in public policy is important as it may provide robust and relevant findings to inform the development and implementation of health policies based on empirical evidence. This ensures that such policies are suitably tailored to address population exigencies (Rudolph et al., 2023). In this vein, the PeNSE data provide a significant opportunity to examine the prevalence of and associated correlation with CyVic.

This study aimed to investigate the potential association of CyVic on emotional distress, substance use, school activities, well-being, and health-related behaviors among Brazilian adolescents using the PeNSE survey. In this study, we hypothesized that (i) girls report more incidences of being cyberbullying victims; (ii) girls report a greater association on mental health indicators, including increased anxiety, depression, irritability, nervousness, and low self-esteem, as a result of CyVic; (iii) CyVic is associated with an increase in substance use as well as earlier onset of substance use; and (iv) CyVic is associated with negative associations on mental health indicators, including increased frequency of suicidal ideation.

## 2 Methods

### 2.1 Ethical considerations

This study used a publicly available secondary database; therefore, it did not require additional approval from our institution’s Research Ethics Committee because it was previously approved by the Brazilian National Health Council (CONEP, n° approval: 3,249,268). The exemption is in accordance with Article 1, paragraph one, item IV of CONEP Resolution 510/2016.

### 2.2 Study design

The PeNSE (National Student Health Surveillance Survey) is a cross-sectional study conducted in Brazil approximately every 4 years since 2009, with subsequent editions in 2012, 2015, and 2019. The 2021 edition was not held due to the COVID-19 pandemic, and the 2024 edition is currently ongoing, with data still unavailable. This manuscript specifically analyzes data from the most recent completed edition (Brazilian Institute of Geography and Statistics (IBGE), 2022). To select participants, PeNSE utilizes a complex, multistage sampling design based on the Brazilian Department of Education’s school registry. This approach allows for the collection of data from a nationally representative sample of students attending both public and private schools across Brazil.

The survey comprises 12 comprehensive domains: (1) General Characteristics, (2) Family Context, (3) Oral Health and Hygiene, (4) Nutrition, (5) Sedentary Behavior and Physical Activity, (6) Tobacco, (7) Alcohol, (8) Other Drugs, (9) Mental Health, (10) Sexual and Reproductive Health, (11) Use of Health Services, and (12) Safety and Violence. Data collection was conducted using self-administered questionnaires, ensuring anonymity and confidentiality. This survey is unique in its ability to compare health indicators across approximately 90 countries because of its alignment with the Global School-Based Student Health Survey (GSHS) offered by the World Health Organization/Center for Disease Control.

This study analyzed the following PeNSE domains: Tobacco, Alcohol, and Other Drugs; Safety and Violence (especially aggression and social isolation); Mental Health; and social relationships at school. Questions related to other domains, such as oral health, were not included as they were not pertinent to the study’s research objectives.

### 2.3 Participants

The PeNSE 2019 study utilized probabilistic, two-stage cluster sampling. In the initial stage, schools (both public and private) were selected based on data from the National School Census (National Institute for Educational Studies and Research (INEP), 2017), with the exclusion of institutions that had fewer than 20 students. Next, classrooms within the selected schools were randomly chosen, and all students in those classrooms were invited to participate.

Students were informed about the study objectives, the voluntary nature of participation, the importance of confidentiality, and their right to withdraw at any time. Those who consented completed a self-administered questionnaire on smartphones, under the supervision of trained IBGE researchers. The questionnaire addressed various topics, including socioeconomic conditions, family context, substance use, violence, and safety.

Initially, PeNSE 2019 aimed to include adolescents based on their school grade, with a total of 159,245 students. However, only those who fully completed the questionnaire (*n* = 158,518) were included in the analysis. Students aged 18 or older (*n* = 11,982) were excluded due to grade repetition. As a result, the final analytical sample comprised 146,536 adolescents aged 13 to 17. Further methodological details are available in specific publications from IBGE.

### 2.4 Measures

Participants were dichotomously categorized based on their responses: *“In the past 30 days, have you encountered threats, derogatory remarks, or humiliation on social media or mobile applications?”* Individuals who responded yes to the question above (*n* = 18,068; 13.33% of the total sample) were categorized as the cyber victim group (CYB), while those who responded no (*n* = 128,468; 87.67% of the total sample) were categorized as the non-cyber victim group (nCYB). Although cyberbullying is a latent variable, the large and representative sample size of over 140,000 respondents provided a solid foundation for understanding its prevalence and associations.

The sociodemographic data collected included Age, Sex, Race, Brazilian Region, Municipality, School Type, School Location, and Living Arrangement (Table 1). The following domains of substance use were considered: (6) Tobacco, (7) Alcohol, (8) Other Drugs, as well as lifetime use of alcohol, cigarettes, and illicit drugs (Table 2). Additionally, we considered the age at first use and the number of days each substance was used within the last month for various substances (Table 3). In the domains of (9) Mental Health and (12) Safety and Violence, data were collected on symptoms of anxiety, depression, stress, social isolation, and whether adolescents had experienced sexual abuse (Table 4).

**Table 1:** Demographic characteristics between adolescents categorized in the cyber victim group (*n* = 18,068; CYB), and non-cyber victim group (*n* = 128,468; nCYB).

	CYB				*n*CYB				*χ* ^2^	*p*	*e*
	*N*	*%*	95% CI		*N*	*%*	95% CI				
			Lower	Upper			Lower	Upper			
Age											
Under 13 y	2,494	10.4	9.6	11.3	22,470	14.5	14.1	14.9	52.87	***	0.03
13 to 15 y	10,453	57.2	55.6	58.7	69,821	54.9	54.3	55.5			
16 or 17 y	5,121	32.4	30.9	33.9	36,177	30.6	30.0	31.1			
Gender									199.4	***	0.08
Girls	11,190	61.9	60.3	63.4	64,096	49.9	49.3	50.5			
Boys	6,878	38.1	36.7	39.7	64,372	50.1	49.5	50.7			
Race									1.59	0.81	0.02
White	6,771	37.0	35.5	38.6	50,910	36.6	36.0	37.1			
Brown	7,842	42.8	41.3	44.4	56,228	43.7	43.2	44.3			
Black	2,095	13.0	12.0	14.0	13,098	12.8	12.4	13.2			
Indigenous	594	3.2	2.7	3.7	3,813	3.2	3.0	3.4			
Asian	766	3.9	3.3	4.5	4,419	3.7	3.5	3.9			
Brazilian region									22.55	***	0.02
North	4,470	11.2	10.4	12.0	27,877	10.0	9.8	10.1			
Northeast	5,983	25.2	24.1	26.3	44,515	27.9	27.7	28.1			
Midwest	2,563	8.6	8.1	9.0	18,282	7.9	7.8	8.0			
Southeast	3,152	41.4	39.8	42.9	23,707	41.2	40.9	41.4			
South	1,900	13.7	12.8	14.6	14,087	13.1	13.0	13.3			
Municipality									0.84	0.36	0.00
Capital/City	9,317	23.3	22.4	24.2	66,012	22.8	22.7	23.0			
Interior/Town	8,751	76.7	75.8	77.6	62,456	77.2	77.0	77.3			
School type									41.04	***	0.03
Private	8,284	14.0	13.4	14.6	65,636	16.4	16.3	16.6			
Public	9,784	86.0	85.4	86.6	62,832	83.6	83.4	83.7			
School location									14.02	***	0.01
Rural	778	6.3	5.5	7.0	6,272	7.8	7.6	8.1			
Urban	17,290	93.7	93.0	94.5	122,196	92.2	91.9	92.4			
Living arrangements									48.52	***	0.05
With neither parent	1,343	7.6	6.8	8.5	7,160	6.2	6.0	6.5			
With one parent	6,989	41.0	39.4	42.6	43,655	36.4	35.8	36.9			
With both parents	9,732	51.4	49.8	52.9	77,624	57.4	56.8	58.0			

% = representative frequency within the population; CI = Confidence Interval; *χ*^2^ = Rao-Scott Chi-square test statistic; p = the significance level; effect = Effect Size as measured by the Cramer’s *V* test (not weighted). ***p < 0.001.

**Table 2:** Crude and adjusted odds ratios for cyberbullying victimization comparing adolescents in the cyber victim group (*n* = 18,068; CYB) and the non-cyber victim group (*n* = 128,468; nCYB) regarding substance use domain.

Variables	Crude				Adjusted			
	OR	95% CI		*p*	OR	95% CI		*p*
		Lower	Upper			Lower	Upper	
Lifetime								
Alcohol				***				***
Yes	2.10	1.94	2.28		1.45	1.32	1.60	
No	REF				REF			
Cigarettes				***				***
Yes	1.96	1.81	2.12		1.26	1.14	1.40	
No	REF				REF			
Illicit drugs				***				0.42
Yes	2.00	1.81	2.20		1.05	0.93	1.20	
No	REF				REF			
Past month								
Has any friend drunk in your presence?				***				***
Yes	2.19	2.04	2.36		1.61	1.48	1.75	
No	REF				REF			
Has any friend used illicit drugs in your presence?				***				***
Yes	2.43	2.23	2.64		1.64	1.48	1.82	
No	REF				REF			

Model is adjusted for age, sex, Brazilian region, type of school (public or private), school location (urban or rural), living arrangements, and all other variables in the table. OR, odds ratio; REF, reference category. ***p < 0.001.

**Table 3:** Differences in substance use between adolescents categorized in the cyber victim group (*n* = 18,068; CYB), and non-cyber victim group (*n* = 128,468; nCYB).

Variables	CYB			n_CYB			*t*	*p*	*e*
	*n*	*M*	*SD*	*n*	*M*	*SD*			
Onset									
Cigarette	4,698	13.10	4.46	19,632	13.35	4.12	−3.43	***	0.06
Alcohol	11,579	12.77	4.30	63,773	13.07	4.24	−7.01	***	0.07
Illicit Substances	2,896	13.60	4.16	11,496	14.14	3.45	−6.45	***	0.14
Frequency of alcohol-related disruptions in relationships, classes, or conflicts.	11,582	0.93	4.74	63,853	0.49	3.73	9.48	***	−0.10
In your life, how many times have you drunk so much that you were really drunk?	11,587	2.22	6.76	63,863	1.66	5.96	8.40	***	−0.09
Days used in the past month									
Cigarettes	4,702	3.39	16.82	19,676	2.35	14.76	3.90	***	−0.07
Alcohol	11,577	2.93	12.15	63,832	1.98	9.97	7.91	***	−0.09
Any drug	2,914	2.06	6.63	11,530	1.60	6.63	3.32	***	−0.07
Cannabis	2,912	1.65	5.09	11,526	1.32	5.03	3.06	**	−0.06
Crack/Cocaine	2,905	0.35	2.95	11,518	0.11	1.73	4.26	***	−0.10

M, mean; SD, standard deviation; *t*, student’s *t*-test for independent samples; *p*, significance level; *e*, effect size measured by Cohen’s *d* test. **p < 0.01, ***p < 0.001.

**Table 4:** Crude and adjusted odds ratios for cyberbullying victimization comparing adolescents in the cyber victim group (*n* = 18,068; CYB) and the non-cyber victim group (*n* = 128,468; nCYB) regarding mental health and safety and violence domains.

Variables	Crude OR				Adjusted OR			
		95% CI		*p*		95% CI		*p*
		Lower	Upper			Lower	Upper	
Past month								
How often did your parent or guardian comprehend your concerns?				***				***
Rarely	2.49	2.26	2.75		1.18	1.04	1.33	
Sometimes	1.59	1.42	1.78		1.12	0.98	1.28	
Most of the time	1.09	0.97	1.22		0.93	0.81	1.06	
Always	REF				REF			
How often did you feel sad?				***				***
Rarely	REF				REF			
Sometimes	1.81	1.63	2.00		1.37	1.21	1.55	
Most of the time	3.68	3.32	4.09		1.72	1.48	1.99	
Always	7.31	6.56	8.14		2.27	1.92	2.69	
How often did you feel that no one cares about you?				***				***
Rarely	REF				REF			
Sometimes	1.89	1.72	2.08		1.26	1.13	1.42	
Most of the time	3.45	3.13	3.81		1.57	1.38	1.80	
Always	5.41	4.92	5.95		1.90	1.65	2.18	
How often did you experience irritation, anger, or a negative mood?				***				***
Rarely	REF				REF			
Sometimes	1.21	1.09	1.34		0.94	0.83	1.05	
Most of the time	2.00	1.80	2.21		1.02	0.90	1.15	
Always	3.36	3.04	3.73		1.15	1.01	1.32	
How often did you sense life’s lack of value?				***				***
Rarely	REF				REF			
Sometimes	2.44	2.22	2.67		1.46	1.30	1.64	
Most of the time	3.67	3.32	4.06		1.58	1.38	1.81	
Always	5.05	4.60	5.53		1.62	1.41	1.86	
How would you rate your health?				***				***
Very good	REF				REF			
Good	1.11	1.01	1.21		0.91	0.82	1.01	
Fair	2.00	1.83	2.19		1.10	0.98	1.22	
Bad	3.22	2.79	3.71		1.25	1.05	1.48	
Very bad	4.98	3.99	6.21		1.68	1.29	2.19	
Have you been abused?				***				***
Not abused	REF				REF			
Abused	3.34	3.07	3.62		2.31	2.11	2.53	

Model is adjusted for age, sex, Brazilian region, type of school (public or private), school location (urban or rural), living arrangements, and all other variables. OR, odds ratio; REF, reference category. ***p < 0.001.

### 2.5 Data analysis

The data analysis was meticulously comprehensive, covering both descriptive and inferential statistics. We initiated the process by scrutinizing the demographic characteristics of the data using a descriptive approach to determine the frequencies and confidence intervals based on the weighted sample. In the realm of inferential statistics, we employed the Rao-Scott Chi-Square Test to analyze categorical variables and compare the main sociodemographic characteristics between the CYB and nCYB groups. The Rao-Scott chi-square test was chosen over the standard chi-square test as it adjusts for complex survey design and weighting, providing more accurate estimates of statistical significance for categorical data in large, stratified samples.

We were transparent in our methodology, sharing our data analysis approach with the scientific community. The magnitudes of the associations were measured using Cramer’s *V* test, considering the number freedom degrees in each analysis (Table 1). We also conducted both crude and adjusted logistic regressions (aOR) (Tables 2, 4) to predict the outcome of CYB, with the adjusted models controlling for the variables based on significant associations in the chi-square test, as shown in Table 1 [age, sex, school location (urban or rural), school type (private or public), and living arrangements]. We minimized potential bias by controlling the logistic regression model with variables that showed significant associations in the chi-square test. We ensured that the model accurately reflected the genuine relationships between the predictors and outcomes, leading to more robust and reliable conclusions.

For the continuous variables (data from Table 3), we analyzed the data distribution using the Kruskal-Wallis test and assessed the homogeneity of variances using Levene’s test. Based on these findings, we conducted a student’s *t*-test for independent samples. Despite the large sample size, our statistical inferences were rigorously validated using normality and homogeneity tests, reducing the chance of obtaining misleading results. Cohen’s *D*-test was used to calculate the effect size, which is particularly suitable for large sample sizes because random sampling variability is less likely to cause fluctuations.

All data analyses were conducted using SAS software 3.81 (Enterprise Edition). The “Survey” package was chosen for its explicit accounting for sample weights, with each value encapsulating the respective individual’s sample weight within the database. This choice was made to ensure the accuracy and reliability of our data analysis, aligning with the best practices in the field.

Following an open science model, we created the following files available on the Open Science Framework: (1) a script containing all the variables analyzed, and (2) two Excel files providing all research results. The spreadsheets contain extensive additional information not included in the tables owing to space restrictions. (3) A template of all tables in the manuscript with each variable coded—the complete study will help readers understand the codes in the spreadsheets and compare them with the same codes in the tables; (4) the complete study database. By providing open access to our data, we aim to facilitate other researchers’ reproducibility and the replication of results. This not only enhances the robustness of our findings, but also opens avenues for the exploration of uncovered findings and the investigation of additional research questions. We look forward to seeing the potential for further research inspired by our open data. All data are available here: https://osf.io/skbpu/.

## 3 Results

### 3.1 Sociodemographic characteristics among CYB e nCYB groups

Of the total sample of adolescents, 13.33% disclosed that they had been victims of cyberbullying during the month preceding the survey, with approximately 60% of them being girls (Table 1). Regarding age, the highest prevalence of CYB was observed among adolescents aged 13–15 years, primarily from the southeastern region of Brazil (a region known for its diverse culture and high population density), who typically live with only one parent. Statistically significant differences were observed across all the demographic characteristics (race and municipality); however, the effect sizes were small.

### 3.2 Substance use domain

Regarding substance use (Table 2), adolescents in the CYB group had higher odds of lifetime substance use, including alcohol, cigarettes, and illicit drugs than those in the nCYB group. This association remained significant even after adjusting for the model (adjusted Odds Ratio – aOR), except for illicit drug use, which lost significance when considering the aOR. Furthermore, the presence of friends who used alcohol or illicit drugs significantly increased the likelihood of adolescents using these substances, regardless of whether they had been victims of cyberbullying or not.

Further analyses considering continuous variables revealed a disturbing trend among adolescents in the CYB group. Not only did they show a greater likelihood of substance use but they also initiated it at an earlier age. The average age during the initiation into cigarette, alcohol, and illicit drug use was significantly lower in the CYB group. These adolescents also reported a higher frequency of alcohol, cigarette, and drug use within the past month, with particular emphasis on crack/cocaine use. The data also indicated that the CYB group experienced more disruptions in their relationships, classes, or conflicts due to alcohol consumption and reported a higher frequency of intoxication episodes.

### 3.3 Mental health and safety and violence domains

As shown in Table 4, the regression models indicated a considerable association between cyberbullying and various mental health issues. The CYB group reported higher odds of loneliness, sadness, irritability, nervousness, and low self-esteem. Additionally, the CYB group showed an increased risk of suicidal ideation and suicide attempts, even after adjusting for sociodemographic and contextual variables. This underscores the serious association of cyberbullying on adolescents’ mental well-being. Adolescents who had experienced sexual abuse were twice as likely to experience cyberbullying.

## 4 Discussion

The present study examined the possible association between CyVic on emotional distress, substance use, school activities, well-being, and health-related behaviors among Brazilian adolescents, using data sourced from the PeNSE. Our cardinal findings indicated that cyberbullying victims were at a greater risk of emotional distress, substance abuse, low levels of health-related behaviors, and school absenteeism. Furthermore, a distinct sex-based trend emerged, with a significantly higher prevalence of CyVic among girls than among boys. The cyber victim cohort also showed a substantively augmented incidence of school absence. These findings substantially support our research hypotheses.

Our findings are consistent with those of previous studies that identified girls’ heightened vulnerability as victims of cyberbullying (Barlett and Coyne, 2023). This increased risk may be due to an increased risk of experiencing indirect forms of cyberbullying such as rumors, defamation, or exclusionary practices, which may be more resistant to preventive efforts (Barlett et al., 2024; García-Fernández et al., 2022). Furthermore, the interplay between sex and CyVic is a multifaceted realm likely to be influenced by many interrelated factors. For example, Viner et al. (2019) explored the interrelationships between social media utilization, mental health, and well-being among approximately 13,000 English adolescents aged 13–16 years. The results indicated elevated levels of social media engagement, CyVic, anxiety, and emotional distress among girls compared to boys. Furthermore, the authors found a mediating role for exposure to cyberbullying and sleep-related complications in the detrimental association of excessive social media usage on girls’ mental health and well-being.

The findings of the elevated prevalence of substance use among cybervictims (CYB group) align with existing research linking CyVic to risky behaviors (Azami and Taremian, 2020; Biagioni et al., 2023). For instance, Azami and Taremian (2020) examined the relationship between conventional bullying victimization, CyVic, substance use, self-harm, and suicide attempts in a high school student population. These findings revealed a robust relationship between CyVic and substance use. This highlights the significance of addressing CyVic as a pivotal risk factor for substance abuse and self-destructive tendencies.

A potential reason for the increased substance use among cyberbullying victims could be that it is a maladaptive coping mechanism for the distress associated with CyVic. Furthermore, instances of cyberbullying may result in anxiety, hopelessness, and low self-esteem (Reed et al., 2015). As such, specific individuals may resort to substances to temporarily alleviate the emotional distress associated with CyVic (Kim et al., 2019; Pichel et al., 2022). The fact that adolescent cyberbullying victims reported heightened instances of sadness, anxiety, and stress in comparison to their nCYB counterparts’ hints at a plausible connection between the psychological fallout of CyVic and the adoption of substance usage as an inadequately adaptive coping strategy (Keum and Ángel Cano, 2023). Wang and Ngai (2022) observed that emotional solace and cyberbullying behavior are often closely related, as cyberbullies may use this tactic to gain dominance or control over others, camouflaging their emotional insecurities. Consequently, adolescent cyberbullies, or cyberbullying victims may have an increased propensity to seek emotional respite through substance use (Keum and Cano, 2021). As expected, CyVic was associated with difficulties outside the virtual realm. The CYB group exhibited markedly elevated levels of aggression, school-related insecurity, and peer and familial violence. These findings suggest that CyVic significantly affects adolescents’ well-being in real life (Chen et al., 2023; Ossa et al., 2023). Although the findings are statistically significant, the effect sizes observed are modest, which is typical in studies with very large sample sizes. Therefore, it is essential to interpret their practical implications carefully. Even small effect sizes can still have meaningful impacts at a population level, especially in representative national surveys such as PeNSE.

This study has various strengths: First, the use of a representative group of Brazilian adolescents has a high generalizability of the findings to Brazilian adults. Second, the study has a multifaceted approach, which investigated CyVic with various correlations, such as emotional distress, substance use, school activities, well-being, and health-related behaviors. Consequently, the present study increases the understanding of the negative associations of CyVic and may help identify avenues for targeted interventions designed to enhance adolescents’ health and well-being.

However, this study has some limitations. First, its cross-sectional design prevents the establishment of causal relationships, and the associations identified may involve complex and bidirectional dynamics. For instance, adolescents who engage in substance use or experience emotional distress may become more vulnerable to online victimization due to increased risk-taking behaviors, lower self-esteem, or impaired social judgment. This suggests a potential bidirectional relationship rather than a strictly unidirectional cause-and-effect link.

Second, cyberbullying victimization (CyVic) was assessed using a single self-report item, which may not fully capture the complexity of cyberbullying experiences and their variability across cultural, social, and geographical contexts (Hasan et al., 2023; Zhu et al., 2022). The operational definition of cyberbullying victimization, based on just one self-report question, might limit the precision in representing the full complexity of this construct, especially regarding its repetitive nature and inherent power imbalances. This limitation could affect the interpretation of our findings. Additionally, the broad scope of PeNSE did not allow for an examination of the different forms or motivations underlying cyberbullying behaviors.

Future research should use longitudinal methods to explore causal pathways between CyVic and negative health outcomes. Implementing refined and standardized measurement tools would enable more accurate comparisons across studies and cultures. Additionally, investigating associations between CyVic, emotional distress, and risky behaviors in different age groups, such as children and young adults, could deepen our understanding of these patterns throughout life.

In conclusion, our findings highlight significant associations between CyVic and emotional distress, substance abuse, unhealthy behaviors, and increased school absenteeism. These issues extend beyond the virtual environment into real-life contexts, including aggression and interpersonal conflicts. Therefore, these results emphasize the need for targeted interventions to mitigate cyberbullying and its harmful effects among adolescents.

## Data Availability

Publicly available datasets were analyzed in this study. This data can be found: https://osf.io/skbpu/.
